# Evaluating Molecular Docking Software for Small Molecule Binding to G-Quadruplex DNA

**DOI:** 10.3390/ijms221910801

**Published:** 2021-10-06

**Authors:** Jonathan Dickerhoff, Kassandra R. Warnecke, Kaibo Wang, Nanjie Deng, Danzhou Yang

**Affiliations:** 1 Department of Medicinal Chemistry and Molecular Pharmacology, College of Pharmacy, Purdue University, 575 W Stadium Ave, West Lafayette, IN 47907, USA; dickerho@purdue.edu (J.D.); kwarneck@purdue.edu (K.R.W.); kbwang@cpu.edu.cn (K.W.); 2 Department of Chemistry and Physical Sciences, Pace University, 1 Pace Plaza, New York, NY 10038, USA; 3 Purdue Center for Cancer Research, Purdue University, 201 S University St, West Lafayette, IN 47906, USA; 4 Purdue Institute for Drug Discovery, Purdue University, West Lafayette, IN 47907, USA; 5 Department of Chemistry, Purdue University, West Lafayette, IN 47907, USA

**Keywords:** G-quadruplex, G4-ligands, docking, scoring, pose prediction, drug design

## Abstract

G-quadruplexes are four-stranded nucleic acid secondary structures of biological significance and have emerged as an attractive drug target. The G4 formed in the *MYC* promoter (MycG4) is one of the most studied small-molecule targets, and a model system for parallel structures that are prevalent in promoter DNA G4s and RNA G4s. Molecular docking has become an essential tool in structure-based drug discovery for protein targets, and is also increasingly applied to G4 DNA. However, DNA, and in particular G4, binding sites differ significantly from protein targets. Here we perform the first systematic evaluation of four commonly used docking programs (AutoDock Vina, DOCK 6, Glide, and RxDock) for G4 DNA-ligand binding pose prediction using four small molecules whose complex structures with the MycG4 have been experimentally determined in solution. The results indicate that there are considerable differences in the performance of the docking programs and that DOCK 6 with GB/SA rescoring performs better than the other programs. We found that docking accuracy is mainly limited by the scoring functions. The study shows that current docking programs should be used with caution to predict G4 DNA-small molecule binding modes.

## 1. Introduction

**Figure 1:**
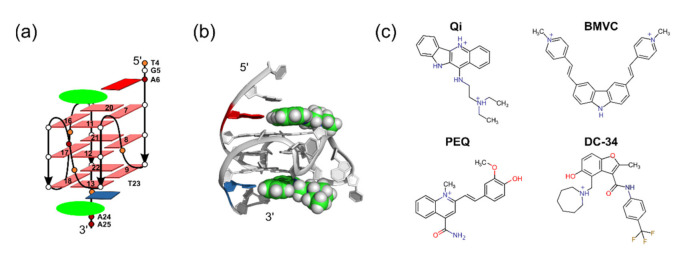
(**a**) Schematic structure of the MycG4 bound by two ligands (green oval) stacking on the external tetrads and recruiting a flanking residue. (**b**) A ribbon representation of the MycG4 in complex with the PEQ ligand bound at the 5′-end and 3′-end (PDB: 2L7V). (**c**) Chemical structures of the four MycG4 ligands studied in this work.

G-quadruplex (G4) is one of the most exciting nucleic acid secondary structures formed in biologically significant guanosine-rich sequences, such as human telomeres, oncogene promoters, replication initiation sites, and 5′- and 3′-untranslated regions (UTR) of mRNA. G-quadruplexes are involved in a number of critical cellular processes, including gene transcription, translation, replication, and genomic stability [1,2,3,4], and have emerged as an attractive new class of molecular targets for drug development [5,6]. They are four-stranded non-canonical secondary structures (Figure 1) formed in DNA or RNA sequences containing consecutive runs of guanosines and form readily under physiologically relevant conditions. Four guanosines associate to form G-tetrads that stack upon each other to form the quadruplex core. In contrast to the linear and uniform DNA duplex, biologically relevant intramolecular G4s are of globular shape and characterized by a vast structural diversity [7].

G4s can be classed as parallel and non-parallel structures depending on the directionality of the involved G-runs, which are connected by loop sequences [1]. In parallel G4s, the 5′- and 3′-terminal flanking regions mostly form capping structures over the external G-tetrads, whereas in non-parallel G4s, the loop regions are also involved in capping structure formation. Parallel G4s are prevalent in the human genome, such as promoter DNA G4s and RNA G4s [3,8]. A prominent example and model structure for parallel G4s with short loops is the major G4 formed in the promoter region of the MYC oncogene, MycG4 (Figure 1a). The MYC protein is an important transcription factor that is commonly deregulated in human cancers; however, it is often considered ‘undruggable’ [9]. Alternatively, the MycG4 is a promising anticancer drug target because of its function as transcription silencer [1,10,11,12,13] and is one of the most studied G4s for small molecule targeting. Structure-activity relationships can guide rational drug development and help identify promising candidates; however, the immense chemical space of possible ligands cannot be fully explored despite the availability of high-throughput screening methods. The time-consuming and work-intensive determination of high-resolution G4-ligand structures is often a formidable task. 

Molecular docking is widely used in rational drug discovery for binding mode prediction, hit identification (virtual screening), and lead optimization [14]. A docking campaign can screen ~10^6^ molecules from a large library against targets over a few days with hit rates as high as 10% and the predicted binding modes for the top ligands often confirmed by subsequent structure determination [15]. It has been estimated that as of 2016, close to 20 drugs have directly benefited from structure-based approaches including docking [14]. Docking can predict the 3D structures of G4-ligand complexes on a large scale in silico and aid the design of small molecules targeting the G4s [16,17,18]. In recent years, docking has also been commonly applied in targeting DNA G4s for hit identification in virtual screening studies or to predict the binding pose of a ligand [16,19,20,21,22,23,24,25,26,27,28,29,30,31]. For example, in a screening of Pt(II)-Phenanthroline complexes with double-stranded DNA and G-quadruplexes, Ang et al. applied AutoDock Vina in combination with biophysical approaches to study the correlations between ligand structure and selectivity for G4 end-stacking over groove-binding [24]. Tomar and coworkers used DOCK 6 with Generalized Born/Solvent Accessible Surface model (GB/SA) rescoring to understand specific stabilizing effects that soy isoflavones exert on G4s [26]. Rocca et al. applied Glide for structure-based virtual screening to identify piperidinyl-amine derivatives that function as dual G4 binders of c-Myc and c-Kit [29].

While docking has been used in a number of studies that targeted G4 DNA, it remains unclear how well docking methods actually captured the experimental G4 binding modes in solution. None of the docking-generated structures of the G4 DNA-ligand complexes in these studies have been experimentally confirmed by structure determination. What is the feasibility of the current docking software for studying small molecular G4 DNA interactions? Most of the docking software programs have been developed for modeling protein-small molecule recognition with scoring functions trained and validated using a large amount of available protein-ligand structures [32,33]. Compared with protein systems, nucleic acids exhibit different physical and chemical properties, including a highly charged backbone that exerts strong electrostatic fields and unique hydration patterns around the solute [34,35]. In addition, G4 DNA possesses unique ligand binding pockets in which stacking interactions play a major role, in contrast to the binding sites found in proteins and many RNA targets. Most ligand binding involves the external G-tetrads with large planar nonpolar surfaces and has fewer opportunities for hydrogen bond formation. All of these unique structural and chemical properties of G4 DNA could present challenges for docking and scoring functions developed primarily for recognizing protein-ligand and RNA-ligand interactions. To date, no systematic validation has been reported on the feasibility of studying small molecular G4 DNA interactions with current docking programs.

In this first evaluation of G4-ligand docking, we focus on the simplest physiologically relevant G4 type, parallel structures with short loops [4], as they should be the least of a challenge for the docking programs. Compared to protein and RNA structures, the available experimental data for G4s are extremely limited. MycG4 is considered a model system for parallel structures and is one of the most studied intramolecular G4s for small molecule binding. However, only eight small molecule-MycG4 complexes have had their solution structures determined by nuclear magnetic resonance (NMR) spectroscopy [36,37,38,39]. We examined the performance in self-docking of four commonly used docking programs, AutoDock Vina, DOCK 6.9 (DOCK 6 in the following), Glide, and RxDock [40,41,42,43,44,45], for reproducing the experimental binding modes using this small dataset consisting of eight small molecule-G4 complexes. These docking programs are academically (AutoDock Vina, DOCK6, and RxDock) or commercially (Glide) available, while DOCK6 and RxDock have been optimized for docking of RNA targets [40,46]. Our results show that DOCK 6 performs better for self-docking of our test molecular systems than other programs; however, the docking success rate for the top-ranked poses is still lower than those typically seen with protein targets [32]. Our analysis indicates that the docking accuracy is limited by the scoring rather than the sampling of the search space. The results also suggest that considering more than the top-scored poses and using independent experimental data- and knowledge-based criteria such as the extent of end stacking may help eliminate false predictions of the top poses. Our study suggests that current docking programs for the accurate prediction of G4 DNA-small molecule binding should be used with caution. 

## 2. Results and Discussion

### 2.1. Using MycG4 as a Model System for Docking Evaluation

Docking of small molecules with G4s is challenging due to the structural plasticity and unusual features of their binding pockets. Intramolecular G4s are considered biologically relevant and can adopt different folding structures, including parallel and non-parallel structures [1]. In parallel G4s with short loops, only the flanking regions at the 5′- and 3′-end cover the external G-tetrads for small molecule interactions (Figure 1a,b). In non-parallel G4s, the loop regions also cover the external G-tetrads, hence small molecule binding can involve both flanking and loop regions. The high plasticity of G4 binding pockets can impede docking studies. Free G4 structures often do not resemble the bound G4 structures because flexible flanking and loop regions adopt different arrangements upon ligand binding, as shown for the binding of epiberberine to a non-parallel telomeric G4 structure [47]. Docking against such a ligand-induced bound conformation needs to consider receptor rearrangement, which is a major challenge and an active area of research [48]. 

Parallel G4s with short loops are the simplest physiologically relevant G4 structures. As illustrated in Figure 1a,b, small molecules bind parallel G4s almost exclusively by stacking on the external tetrads at both the 5′- and 3′-end (end-stacking). Compared to other G4s, the ligand recognition is simpler because only the flanking regions are involved in ligand interactions, which results in a large binding pocket that can accommodate diverse small molecules. Often, the ligand recruits the adjacent flanking residue to form a DNA-ligand joint-plane that maximizes stacking interactions with the tetrad and allows for specific interactions with DNA by anchoring the ligand (Figure 1b) [36,37,38,39]. Therefore, stacking interactions are significantly more important for G4 binding than for the recognition of proteins or alternative RNA structures, which have been used previously to train and evaluate the scoring functions of docking programs [40,41,42,43,44,45,46].

Compared to protein and RNA structures, the available experimental data for G4s are extremely limited. Although MycG4 is the most studied system for parallel G4s with short loops [10], it only has four small molecule complex structures determined in solution. Four small molecules, i.e., quindoline I (Qi), BMVC, PEQ, and DC34, bind the parallel MycG4 with a 2:1 stoichiometry (Figure 1c) [36,37,38,39]. This provides a total of eight small molecule complexes with MycG4. In this study, we focused on evaluating docking software for the simplest self-docking to bound G4 DNA structures, which does not involve receptor reorganizations. Notably, we showed recently that these four ligands recognize the MycG4 in a conserved way, which is an important property for successful docking studies as it suggests that other molecules likely bind in a similar way without considerable disturbance of the bound G4 conformation [37]. 

### 2.2. Docking Study Using Four Commonly Used Docking Programs

We examined four docking programs, i.e., AutoDock Vina, DOCK 6, Glide, and RxDock, for whether and how well they can reproduce the experimental results in silico by self-docking using the eight MycG4-ligand complex structures. RxDock has not been used with G4s previously; however, it has been optimized for docking of RNA targets [40]. The docking pose is considered experimental-like if the ligand root-mean-square deviation (RMSD) between the docked pose and the experimental NMR structure is less than 2.5 Å. 

#### 2.2.1. AutoDock Vina

**Figure 2:**
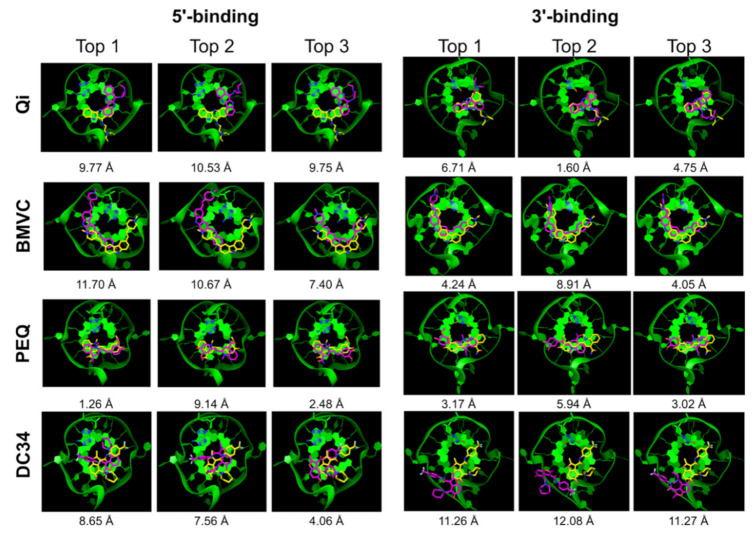
AutoDock Vina top 3 docked poses (purple) superimposed onto the NMR poses (yellow). The G4 DNA is shown as green ribbon.

We first tested AutoDock Vina [41] for docking small molecules to MycG4. AutoDock Vina is a successor of AutoDock [49]. The program uses iterated local search, which involves a succession of steps of mutations and local optimizations. The scoring function consists of intermolecular and intramolecular contributions and was tuned using the PDBbind database [50], which is a collection of experimental binding affinity data for protein-ligand complexes. Energy grids were created by manually centering and defining the dimensions of a grid box to include the receptor site, which was defined herein as the 5′- or 3′- tetrad and flanking nucleotides. The results are summarized in Table 1. Of the eight G4-ligand complexes tested, the experimental binding poses were only successfully sampled in four cases. Moreover, the 5′-end PEQ complex was the only experimental pose correctly ranked at the top (success rate 12.5%). Even if we defined an experimental pose within the top 5 conformers as a success [51], the docking success rate was just 25% (2 out of 8). A superposition of the best three docked poses for each binding site with the experimentally derived poses shows their differences (Figure 2). Overall, our result indicates that both the sampling and scoring of AutoDock Vina need to be improved for modeling G4-small molecule recognition.

#### 2.2.2. DOCK 6

**Figure 3:**
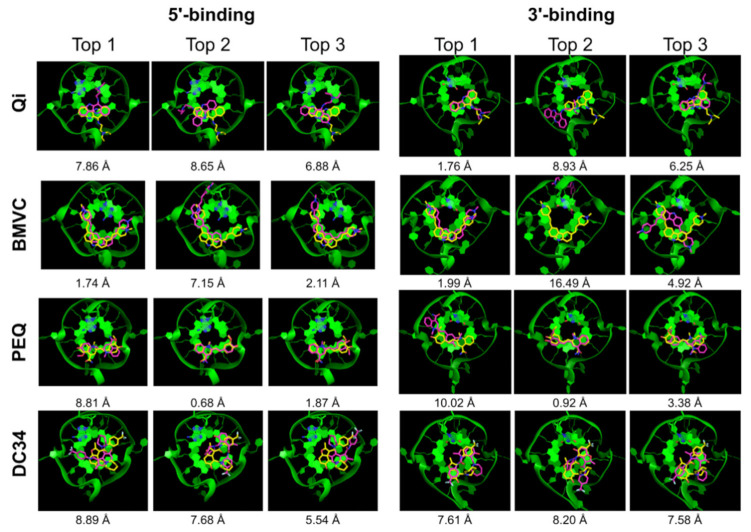
DOCK 6 top 3 docked poses (purple) superimposed onto the NMR poses (yellow). The G4 DNA is shown as green ribbon.

We next performed docking on the same eight complexes of small molecules and MycG4 using DOCK 6 with and without GB/SA rescoring. The DOCK 6 program uses an anchor-and-grow approach to sample the search space. Different parameters for sphere generation were tested to optimize the mapping of the unique G4 binding pockets. The best results were obtained with a maximum sphere radius of 5 Å and a minimum sphere radius of 1.4 Å. The 6-12 Lennard-Jones potential was used for Van der Waals term. Ligand poses were first ranked and clustered based on the default grid-based score used by DOCK 6. The results are summarized in Table 2, and the corresponding docked conformations are shown in Figure 3. DOCK 6 successfully yielded experimental-like poses sampled in seven of eight complexes (Table 2, All), exhibiting good sampling power. Using the default grid-based scoring function, DOCK 6 correctly ranked the experimental poses at the top 1 for three out of eight complexes (Table 2, Top 1), a success rate of 37.5%. Notably, while DOCK 6 exhibited a moderate top 1 docking success rate, the top 5 docked poses showed a significantly higher success rate of 75% in all but the two DC34 complexes (Table 2, Top 5). This result indicated that the correct pose could be found among the top 5 poses with a high probability by DOCK 6. Interestingly, for the 3′-end PEQ complex, the second-best docked pose had an RMSD of 0.92 Å and showed more extensive stacking interactions with the external G-tetrad as compared with the top pose, which had a much worse RMSD of 10.02 Å (Figure 3). This result suggests that the degree of stacking could be used as an additional filter to eliminate clearly incorrectly ranked top poses.

**Figure 4:**
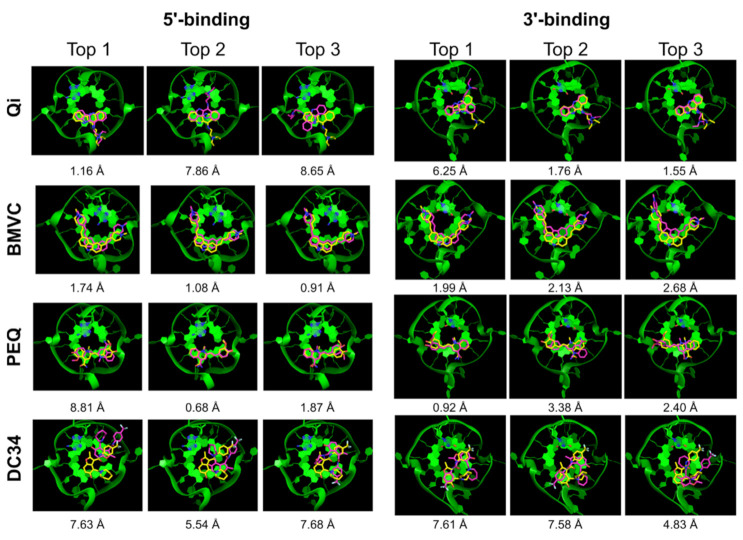
DOCK 6 rescored using GB/SA. Top 3 docked poses (purple) superimposed onto the NMR poses (yellow). The G4 DNA is shown as green ribbon.

GB/SA scoring is designed to provide a better treatment for desolvation effects. We also tested whether GB/SA (GB^HCT^) rescoring of the docked poses improves the pose ranking in DOCK 6 (Table 2, Figure 4) [52,53]. The GB/SA rescoring in DOCK 6 improved the top 1 ranking success rate to 50%, with correct top 1 ranking achieved in four out of eight complexes. Specifically, the rescoring improved the ranking for the 5′-end Qi complex and 3′-end PEQ complex, in which the experimental-like pose was correctly placed at the top (Table 2). However, the GB/SA rescoring did not improve the ranking of the 5′-end PEQ complex and actually displaced the correct pose from top 1 for the 3′-end Qi complex.

#### 2.2.3. Glide SP

**Figure 5:**
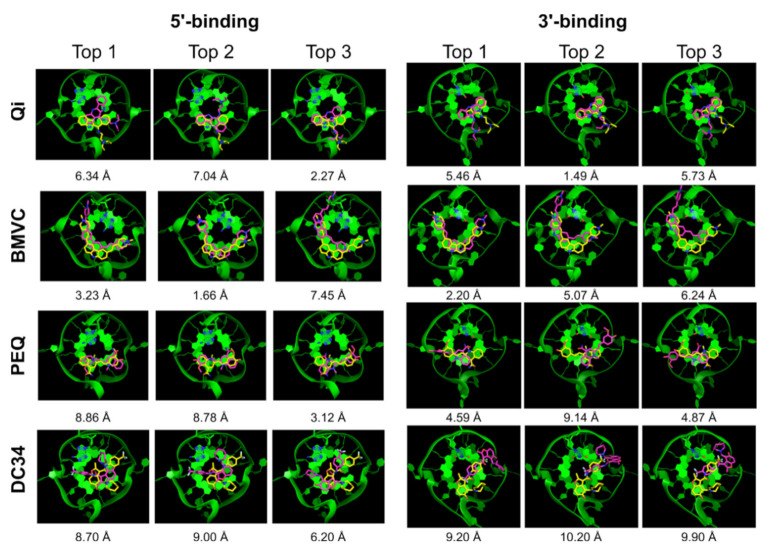
Glide SP top 3 docked poses (purple) superimposed onto the NMR poses (yellow). The G4 DNA is shown as green ribbon.

We next performed a docking study using Glide SP in the Schrödinger’s Maestro interface. The SP protocol is used in pose prediction. Table 3 summarizes the Glide SP docking results. Glide also showed good sampling power and the experimental poses were found in the majority of the complexes (6 out of 8). The top 1 docking success rate was low, with only one complex correctly ranked (12.5%) (Table 3, Top 1). However, the top 5 success rate was much higher with the experimental-like poses ranked in the top 5 poses in five out of eight complexes (62.5%). Figure 5 shows the top 3 Glide docked structures superimposed onto the NMR structures. Again, additional criteria such as the degree of end stacking may help filter out incorrect top ranked poses in some cases. For example, the second-best scored pose of the 5′-end BMVC complex (RMSD = 1.66 Å) showed better G4 stacking than the top scored pose (RMSD = 3.23 Å).

Similar to DOCK 6, we tested the Prime MM-GBSA in the Schrodinger Inc package to rescore the Glide SP docked poses. Only in one case, the 5′-end PEQ complex, MM-GBSA improved the ranking of an experimental-like pose (RMSD = 0.4 Å) from top 4 to top 1.

#### 2.2.4. RxDock

**Figure 6:**
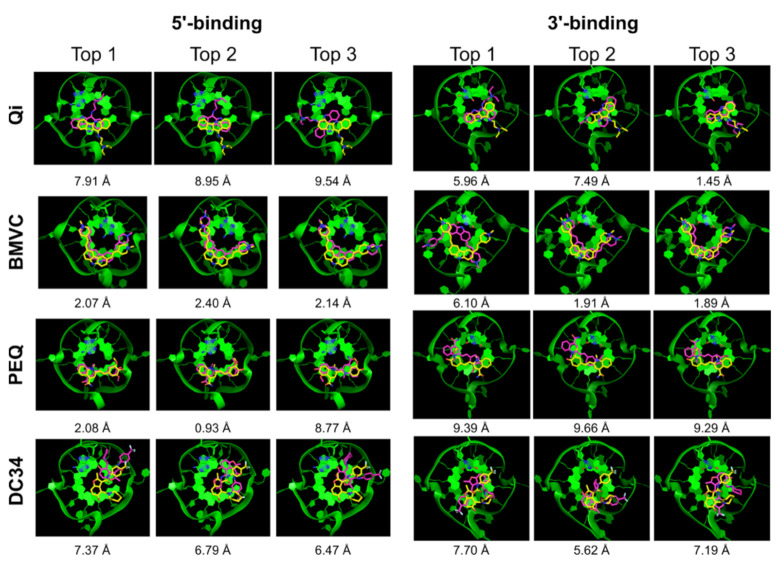
RxDock top 3 docked poses (purple) superimposed onto the NMR poses (yellow). The G4 DNA is shown as green ribbon.

We also performed docking using RxDock, a derivative of the rDock program [40]. Since RxDock was developed for RNA, adjustments were needed to work with DNA as described in the Materials and Methods section. The docking cavity was defined using a radius of 12 Å and a grid spacing of 0.5 Å around the reference 5′- or 3′-ligand using default parameters. One hundred ligand poses were generated and scored using the SF5 scoring function, which includes a desolvation potential and showed the best performance for RNA among the implemented functions [40]. The results of the RxDock docking are shown in Table 4. RxDock also showed the same good sampling power as DOCK 6, with the experimental-like poses sampled in seven out of eight cases. However, the scoring results were more comparable to Glide SP. Only two of the correctly sampled experimental poses were ranked at top 1, resulting in a top 1 docking success rate of 25%. Including the top 5 poses improved the docking success rate to 62.5% (in five out of eight complexes). The docking results are shown in Figure 6. Once again, we found that using the criteria of optimal stacking could in some cases help eliminate false top binding mode: for example, the top pose of BMVC in the 3′-end complex (RMSD of 6.10 Å) showed poor stacking with the 3′-tetrad and could be rejected on this basis.

### 2.3. Comparison of the Four Docking Programs

Table 5 summarizes the self-docking results of eight small molecule-MycG4 complexes using the four different docking programs. Except for AutoDock Vina, the programs could rather successfully sample the structural space of the tested ligands and generate the correct pose. AutoDock Vina was the least successful program for docking small molecules to G4s. 

Overall, DOCK 6 performed best in scoring among the four programs (Table 5). While DOCK 6 exhibited a low top 1 docking success rate (37.5%), using GB/SA rescoring improved it to a moderate success rate of 50%. However, the correct ranking rate of the top 1 pose was still lower compared with protein-ligand docking, where the experimental poses are often scored in the top positions (success rate 60–90%) [32,33]. Clearly, for the G4 DNA-ligand docking, the docking success rate was hampered by the limitations in the scoring function. The available scoring functions were never trained against G4 DNA systems, thus the experimental poses were poorly ranked. In addition, an increase in NMR complex structures (a larger data set) could help the future docking and evaluation.

Encouragingly, including the top 5 ranked poses significantly improved the docking success rate for DOCK 6 (75%), and for Glide and RxDock (62.5%) (Table 5). A notably worse performance was observed for AutoDock Vina and the DC34 ligand. This suggests that there is a reasonably high probability to find the correct pose among the top 5 poses and that applying knowledge of the features of G4-small molecule interactions might offer a way for its identification [51,54]. The correct pose might be identified by excluding better-scored wrong poses based on chemical reasoning. For example, extensive stacking interaction is a hallmark of G4 ligands, and the top-ranked poses that do not fulfill this requirement could be excluded. Of note, in about half of the incorrectly predicted poses, the ligand failed to maintain adequate stacking interaction with the external G-tetrad. For example, in the docked structures for the 3′-end PEQ complex by DOCK 6, the second best pose (RMSD = 0.92 Å) showed more extensive stacking interactions with the external G-tetrad as compared with the top pose (RMSD = 10.02 Å) (Figure 3). This was also observed for the BMVC complexes in the docked structures by Glide and RxDock (Figure 5 and Figure 6). Thus, using the degree of stacking as an additional filter may help eliminate clearly incorrectly ranked top poses. 

**Figure 7:**
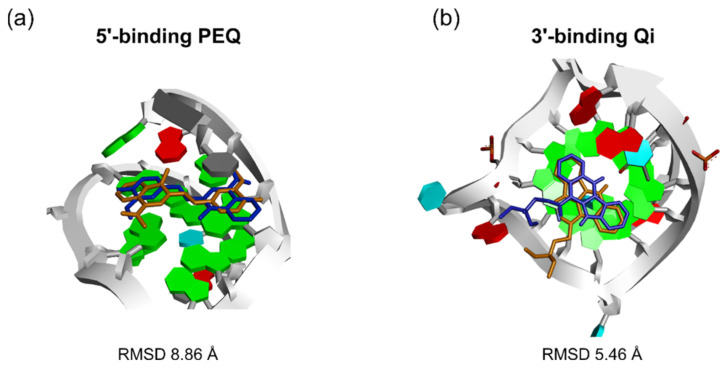
Examples of top poses from Glide SP (blue) in which the ligand was flipped 180° relative to the NMR poses (orange). (**a**) Top scored pose of 5′-end PEQ complex superimposed onto the NMR pose. (**b**) Top pose of 3′-end Qi complex superimposed onto the NMR pose.

However, it is noted that this approach is not without risk. It will not work when the incorrectly ranked top pose shows good end stacking with the G4 but with an incorrect orientation (Figure 7). Examining the complexes for which the Glide SP incorrectly assigned the top poses, we found that in some cases while the polycyclic ring systems of the ligands were able to form extensive stacking interactions with the end G-tetrad plane, the ligands were flipped 180° relative to the NMR poses. For example, the top 1 pose for the 5′-end PEQ complex (RMSD = 8.86 Å) differed from the NMR pose by only a rotation around the ethenyl linker that changes the binding orientation without altering the stacked surface of the G-tetrad (Figure 7a). A similar phenomenon was also observed for the top 1 pose in the 3’-end Qi complex (RMSD = 5.46 Å) (Figure 7b). In such cases, using the degree of stacking alone as a filter will not be able to eliminate these two false predictions, which would require a more accurate energy model.

## 3. Materials and Methods

### 3.1. Structure Files

The structure files for the MycG4 in complex with Qi (PDB: 2L7V), BMVC (6O2L), PEQ (7KBX), and DC34 (5W77) were downloaded from the Protein Data Bank and used to create the input files as described for every docking software program. Only the first structure of the NMR structural ensembles was used. The final figures were generated using PyMol [55].

### 3.2. AutoDock Vina

AutoDock Vina [41] is the successor of AutoDock [49] and was downloaded under vina.scripps.edu (accessed on 5 March 2020). The receptor was exported as a pdbqt file using AutoDock Tools 1.5.6 [49]. The ligand was exported as a pdbqt file using AutoDock Tools after defining the rotatable bonds. Docking was performed in triplicate using a maximum mode number of 50, exhaustiveness of 10, and energy range of 5 to perform an exhaustive sampling of the search space as described previously [56].

### 3.3. DOCK 6

DOCK 6 was downloaded under dock.compbio.ucsf.edu/DOCK_6 (accessed on 26 June 2020). Input files were created with the Chimera software [57]. To prepare the system for docking, charges based on the Amber force field were assigned to the G4s, and AM1-BCC charges were calculated for the ligands. Different parameters for sphere generation were tested to optimize the mapping of the unique G4 binding pockets, and the best results were obtained with a maximum sphere radius of 5 Å and a minimum sphere radius of 1.4 Å. Spheres in a 10 Å radius of either the 5′- or the 3′-bound ligands from the experimentally determined structures were selected to define the box specifying the boundaries of each binding pocket. The energy grids were set up using a grid spacing of 0.3 Å and a 6–12 Lennard-Jones potential [52,53]. Ligand poses were ranked and clustered based on the default grid-based score used by DOCK 6. In addition, a Hawkins GB/SA rescoring of the docked poses was performed with grid-based electrostatic and van der Waals values. The symmetry-corrected RMSD (RMSDh) implementation in DOCK 6 was used for BMVC due to the high symmetry of BMVC that was not taken into account by the standard RMSD calculation [58,59].

### 3.4. Glide

The Glide docking was performed using the Schrödinger’s Maestro interface (Schrödinger, LLC, NY, USA). In Glide, the docking hierarchy starts with a systematic conformational expansion of the ligand, followed by placement in the receptor site. Minimization of the ligand in the field of the receptor is then carried out using the OPLS3 force field with a distance-dependent dielectric. The lowest energy poses are then subjected to a Monte Carlo procedure that samples nearby torsional minima. The best pose for a given ligand is determined by the composite Emodel score. GLIDE has a set of three choices for default docking behavior: standard-precision (SP), high-throughput virtual screening (HTVS), and extra-precision (XP) docking. In XP, sampling is more extensive, using the results from SP docking as a starting point for a high-resolution anchor-and-grow strategy. The XP scoring function contains several additional terms beyond those present in GlideScore, including terms for hydrophobic enclosure and large desolvation penalties.

### 3.5. RxDock

RxDock is derived from the rDock program [40] and was downloaded under www.rxdock.org (accessed on 24 May 2020). All input files were created using PyMol [55]. Since RxDock was developed for RNA, adjustments were needed to work with DNA. Specifically, the RNA entries in the data/sf/RbtIonicAtoms.prm file found in the RxDock installation directory were copied and renamed to DA, DG, DC, and DT. One hundred ligand poses were generated by three stages of genetic algorithm search with subsequent low temperature Monte Carlo and simplex minimization steps. They were scored using the SF5 scoring function, which includes a desolvation potential and showed the best performance for RNA among the implemented functions [40]. Because RxDock does not cluster similar poses, the 100 docked output poses were clustered using Open Babel and a cluster radius of 1.5 Å [60].

## 4. Conclusions

Although G4 DNA has been an important target in structure-based anticancer drug discovery, no studies have validated commonly applied docking methods against G4s. In this work, we sought to fill this gap by evaluating the accuracy of four widely used docking programs in reproducing the experimental binding modes of ligands bound to a simple G4 DNA system, the parallel stranded G4 with short loops. The NMR structures were based on experimental data and should represent the main features of ligand recognition independent of their age. Therefore, these features should be reproducible by an appropriate docking program. Although the limited structural data of G4 DNA compared to protein or RNA structures did not allow for an exhaustive analysis, this case study provided insights into the feasibility of current docking programs to examine G4-ligand interactions. Encouragingly, most programs sampled the search space efficiently and often the experimental poses were found among the best five poses. However, the correct scoring was more challenging for these G4-ligand systems, with best results obtained from DOCK 6. DOCK 6 with GB/SA seems to be able to predict the pose of small molecules that bind to parallel G4s. The challenge could be greater with more complex non-parallel G4 structures. This result is not surprising, since the scoring functions used in the current docking programs are calibrated for binding of small molecules to protein or RNA receptors. Therefore, we hope that this study can motivate future developments of improved scoring functions for G4 binding, as well as stimulate the experimental determination of more high-resolution G4 structures in complex with small molecules, which are needed to train scoring functions. In conclusion, we recommend considering and performing docking of small molecules to G4 DNA with caution. Chemical reasoning might allow one to better identify the experimental pose among the best scored poses; however, virtual screening of a large library might not be feasible for G4 DNA at this time.

## Figures and Tables

**Table 1:** Results obtained using AutoDock Vina.

Ligand	5′-Complex					3′-Complex				
	Correct Pose			Pose RMSD		Correct Pose			Pose RMSD	
	All	Top 1	Top 5	Top	Best	All	Top 1	Top 5	Top	Best
Qi	x	x	x	9.77	5.34	√	x	√	6.71	1.60
BMVC	√	x	x	11.70	1.98	x	x	x	4.24	3.23
PEQ	√	√	√	1.26	1.26	x	x	x	3.17	2.64
DC34	√	x	x	8.65	2.41	x	x	x	11.26	11.26

**Table 2:** Results obtained using DOCK 6 ^1^.

Ligand	5′-Complex					3′-Complex				
	Correct Pose			Pose RMSD		Correct Pose			Pose RMSD	
	All	Top 1	Top 5	Top	Best	All	Top 1	Top 5	Top	Best
Qi	√	x	√	7.86	1.16	√	√	√	1.76	1.55
*GB/SA*	-	√	√	1.16	1.16	-	x	√	6.25	1.55
BMVC	√	√	√	1.74	0.91	√	√	√	1.99	1.30
*GB/SA*	-	√	√	1.74	0.91	-	√	√	1.99	1.30
PEQ	√	x	√	8.81	0.68	√	x	√	10.02	0.92
*GB/SA*	-	x	√	8.81	0.68	-	√	√	0.92	0.92
DC34	x	x	x	8.89	3.63	√	x	x	7.61	1.85
*GB/SA*	-	x	x	7.63	3.63	-	x	x	7.61	1.85

^1^ Using the 6-12 Lennard-Jones potential for Van der Waals term.

**Table 3:** Results obtained using Glide SP.

Ligand	5′-Complex					3′-Complex				
	Correct Pose			Pose RMSD		Correct Pose			Pose RMSD	
	All	Top 1	Top 5	Top	Best	All	Top 1	Top 5	Top	Best
Qi	√	x	√	6.34	2.27	√	x	√	5.46	1.49
BMVC	√	x	√	3.23	1.16	√	√	√	2.20	2.20
PEQ	√	x	√	8.86	0.40	x	x	x	4.59	4.11
DC34	x	x	x	8.70	6.20	√	x	x	9.20	1.98

**Table 4:** Results obtained using RxDock.

Ligand	5′-Complex					3′-Complex				
	Correct Pose			Pose RMSD		Correct Pose			Pose RMSD	
	All	Top 1	Top 5	Top	Best	All	Top 1	Top 5	Top	Best
Qi	√	x	x	7.91	1.52	√	x	√	5.96	0.85
BMVC	√	√	√	2.07	1.42	√	x	√	6.10	1.16
PEQ	√	√	√	2.08	0.93	√	x	x	9.39	0.38
DC34	x	x	x	7.37	3.54	√	x	√	7.70	1.48

**Table 5:** Overall results of small molecule docking to MycG4 using different docking software ^1^.

Ligand		Correct Pose				Correct Pose Top1					Correct Pose Top5				
		Auto Dock Vina	Dock 6	Glide	RxDock	Auto Dock Vina	Dock 6	Dock 6 GB/SA	Glide	RxDock	Auto Dock Vina	Dock 6	Dock 6 GB/SA	Glide	RxDock
Qi	5′	x	√	√	√	x	x	√	x	x	x	√	√	√	x
	3′	√	√	√	√	x	√	x	x	x	√	√	√	√	√
BMVC	5′	√	√	√	√	x	√	√	x	√	x	√	√	√	√
	3′	x	√	√	√	x	√	√	√	x	x	√	√	√	√
PEQ	5′	√	√	√	√	√	x	x	x	√	√	√	√	√	√
	3′	x	√	x	√	x	x	√	x	x	x	√	√	x	x
DC34	5′	√	x	x	x	x	x	x	x	x	x	x	x	x	x
	3′	x	√	√	√	x	x	x	x	x	x	x	x	x	√

^1^ A check mark represents an experimental-like pose (RMSD < 2.5 Å from the NMR structure). A cross mark stands for a non-experimental-like pose (RMSD > 2.5 Å from the NMR structure). The “Correct Pose” columns show whether an experimental-like pose is sampled regardless of its score. The Top 1 and Top 5 columns show whether an experimental-like pose is scored at top 1 or within top 5 poses. Green color shading indicates a correctly represented experimental-like pose, and red color shading indicates an incorrectly represented pose.
